# Consistency and factorial invariance of the Davidson trauma scale in heterogeneous populations: results from the 2010 Chilean earthquake

**DOI:** 10.1002/mpr.1516

**Published:** 2016-07-25

**Authors:** Bryan Kevan

**Affiliations:** ^1^ Federal Reserve Board Washington DC USA

**Keywords:** methodology, PTSD, scale validation, trauma

## Abstract

This investigation seeks to validate an application of a standardized post‐traumatic stress symptom self‐report survey, the Davidson Trauma Scale (DTS), with a large, heterogeneous population of earthquake victims. While previous studies have focused primarily on small samples, this investigation uses a unique dataset to assess the validity of this application of the DTS while accounting for heterogeneity and sample size. We use concurrent validity and reliability analysis tests to confirm the validity of the scale. Further, confirmatory factor analysis is used to test the fit of the data's factor structure against previously established trauma models. Finally, these fit tests are repeated across different mutually exclusive vulnerability subsets of the data in order to investigate how the invariance of the scale is affected by sample heterogeneity. We find that this particular application of the scale is, on the whole, reliable and valid, showing good concurrent validity. However, evidence of variability is found across specific vulnerability subsets, indicating that a heterogeneous sample can have a measurable impact on model fit. © 2016 The Authors International Journal of Methods in Psychiatric Research Published by John Wiley & Sons Ltd

## Introduction

Assessing the incidence of psychological trauma symptoms in large populations through non‐clinical means is an understandably difficult task. Yet, when economically and socially heterogeneous populations suffer through a large single exogenous stressor, such as a natural disaster, it is important to have a way to quickly and accurately assess the incidence of post‐traumatic stress disorder (PTSD) symptoms in affected populations. The gathering of clinical data that can offer a diagnosis of PTSD is a frustratingly costly and imperfect science. Clinician‐administered symptom assessment scales are the most widely‐used alternative to rigorous clinical interview‐based diagnoses, but another cheaper alternative is the use of post‐traumatic stress symptom self‐report surveys, in which respondents report symptoms themselves (Elhai *et al.*, 2005).

One commonly used method in large‐scale post‐traumatic stress symptom assessment is the self‐report survey of traumatic disease called the Davidson Trauma Scale (DTS), proposed by Davidson *et al.* (1997). This particular scale has been studied at length in different languages and settings, and with different stressors (Davidson *et al.*, 1997; King *et al*., 1998b; Palmieri *et al.*, 2007; Seo *et al*., 2008; McDonald *et al*., 2009; Leiva‐Bianchi and Araneda, 2013). In each study, the scale's internal consistency and reliability has held up remarkably well to a wide array of validity assessments. Yet, most of these applications involve relatively small, homogenous samples. One open area of research involves the application of the scale and the measurement of post‐traumatic stress symptoms across particularly large or heterogeneous populations. In particular, not much research has focused on how the validity and reliability of the scale are affected by an application across a large population with many different levels of economic volatility or physical exposure.

This paper investigates these issues, using DTS data from a survey administered to a large, Spanish‐speaking and heterogeneous 22,000‐household sample of victims of the 2010 8.8 magnitude earthquake off the coast of Concepcion, Chile.

Four principal methodologically‐motivated research questions are examined:

1. Can this Chilean earthquake data be used to confirm previously established DTS factor structures?
2. Once a recognizable factor structure's existence is confirmed, does it display good structural invariance across subsets of the population with different levels of economic volatility, poverty, and physical exposure?
3. Does this scale display good concurrent validity with characteristics associated with traditional trauma risk factors?
4. Is the application of the scale reliable as a whole and across the heterogeneous subsets?

The *Diagnostic and Statistical Manual of Mental Disorders, Fourth Edition* (DSM‐IV; American Psychiatric Association, 2000) is a comprehensive volume that describes all recognized mental disorders and their symptoms. PTSD, for example, is listed in the manual as an anxiety disorder, with a large number of details on the disorder's risk factors, symptoms, and treatments. PTSD is a diagnosable anxiety disorder in the DSM‐IV, but its actual nuances and psychological complexities are hard to diagnose or describe without clinical work. Self‐rating scales may be able to predict a future PTSD diagnosis by assessing certain symptoms, but without an actual clinical diagnosis it is impossible to truly capture the full complexities of the diagnosable disorder on an individual level. Symptom self‐report scales take what is published in the DSM‐IV and try to, for the purpose of large‐scale research or future clinical work, predict where a diagnosis might occur (Taylor *et al.*, 2001). The DSM‐IV contains three criteria for measuring four symptom clusters (intrusions, avoidance, numbing, and hyperarousal) of trauma symptoms following a traumatic event.

Table 1 lists the 17 questions that make up the DTS survey structure. Each question is asked twice, once for frequency and once for intensity. But how well does the DTS serve as a predictor of an eventual PTSD diagnosis? Davidson's earliest study (Davidson *et al.*, 1997) found a threshold of 40 (out of 136) to be a good predictor (83% diagnostic efficiency) of a PTSD diagnosis. Later diagnostic efficiency studies found different thresholds that offer an acceptable 80–85% diagnostic efficiency. McDonald *et al.* (2014) advocates a cutoff score of 68 to 72, Sijbrandij *et al.* (2008) advocates a cutoff of 64, Seo *et al.* (2008) advocates a cutoff at 67, and Chen *et al*. (2001) advocates a cutoff at 44. Convergent validity has also confirmed the scale's validity against other more rigorous symptom scales and assessments, including those given by clinicians. For example, high correlations were found between the scale and other clinician‐ and self‐administered scales, such as the Clinician‐adminstered PTSD Scale (CAPS) (Davidson *et al.*, 1997), and anxiety elements of the self‐report Symptom Checklist‐90‐R (SCL‐90‐R) (McDonald *et al*., 2009). While those assessments were done in English, the Spanish version of the DTS has also shown acceptable convergent validity with the CAPS and Treatment Outcome PTSD Scale‐8 (TOP‐8) (Bobes *et al*., 2000).

**Table 1:** Davidson Trauma Scale (DTS) items and proposed factor models

		Models		
Scale Item	Question	DSM‐IV Symptoms	Simms *et al*. (2002)	King DW *et al*. (1998)
1	Have you had painful images, memories or thoughts of the event?	B	I	R
2	Have you had distressing dreams of the event?	B	I	R
3	Have you felt as though the event was re‐occurring?	B	I	R
4	Have you been upset by something which reminded you of the event?	B	I	R
5	Have you been avoiding any thoughts or feelings about the event?	C	I	A
6	Have you been avoiding doing things or going into situations which remind you about the event?	C	A	A
7	Have you found yourself unable to recall important parts of the event?	C	A	N
8	Have you had difficulty enjoying things?	C	D	N
9	Have you felt distant or cut off from other people?	C	D	N
10	Have you been unable to have sad or loving feelings?	C	D	N
11	Have you found it hard to imagine having a long life span fulfilling your goals?	C	D	N
12	Have you had trouble falling asleep or staying asleep?	D	D	H
13	Have you been irritable or had outbursts of anger?	D	D	H
14	Have you had difficulty concentrating?	D	D	H
15	Have you felt on edge, been easily distracted, or had to stay 'on guard'?	D	D	H
16	Have you been jumpy or easily startled?	D	H	H
17	Have you been physically upset by reminders of the event?	C	H	R

Note: In DSM‐IV, letters signify symptom categories. In Simms *et al*. (2002) I = Intrusive, A = Avoidance, D = Dysphoria, and H = Hyperarousal. In King *et al*. (1998) R = Re‐experiencing, A = Avoidance, N = Numbing, H = Hyperactivity

In Table 1, the column labeled “DSM‐IV Symptoms” indicates the criteria designed by the DSM‐IV that each item is intended to measure. Yufik and Simms (2010) provide a meta‐analysis of almost 40 of these factor exploration papers. These papers use a variety of scales, with differing methodologies but all based on the same 17 symptom questions as in the DSM‐IV. The three columns to the right in Table 1 show the factor analyses of the applications of the scale used in the labeled studies (DSM‐IV; King *et al.*, 1998a; Simms *et al.*, 2002). These are the groupings of variables that have been identified by Yufik and Simms' (2010) meta‐analysis as the most common factor structures explored and confirmed in previous literature. Davidson's original factor analysis on a combined sample of war veterans, rape victims and hurricane victims, could not distinguish between avoidance and numbing categories, giving only a two‐factor solution in the overall 353‐person sample and a difficult to interpret six‐factor solution in the extremely small 67‐person sample of those over a diagnostic threshold. He compared these to the proposed four‐factor solution of the DSM‐III (the published version of the DSM at the time) and found similarities, but did not go so far as to propose a firm grouping. Note that Table 1 lists factors as they were originally labeled in each respective paper. This naming of the factors was subject to the researcher's own interpretation of results, and was often based on how well their factors aligned with the DSM‐IV and previous factor analyses.

The two other factor groupings provide two different four‐factor solutions. In particular, the four‐factor solutions advocated by King *et al*. (1998a) and supported by further research (Asmundson *et al.*, 2000; Asmundson, 2004; DuHamel *et al.*, 2004; McWilliams *et al.*, 2005; Palmieri and Fitzgerald, 2005). King *et al.* (1998a) used a sample of 524 mixed‐gender treatment‐seeking Vietnam veterans and a 17‐item clinician‐administered scale similar to the DTS. The results of his factor analysis are in the last column of Table 1. Larger sample sizes, and differentiated sources of victims may have led to this increased ability to distinguish between factors. Asmundson *et al.* (2000) used a clinical sample of 349 primary care patients, DuHamel *et al.* (2004) a clinical sample of 236 cancer survivors, McWilliams *et al.* (2005) a clinical sample of 429 people with a general history of PTSD, and Palmieri and Fitzgerald (2005) a sample of 1218 workplace sexual harassment victims.

An alternate four‐factor solution, very different from the DSM‐IV structure, was proposed by Simms *et al.* (2002) and has been supported often in further research (Baschnagel *et al.*, 2005; Boelen *et al.*, 2008; Elklit and Shevlin, 2007; Krause *et al.*, 2007; Milanak and Berenbaum, 2009). The resulting structures from these four‐factor solutions, shown in the second column of data in Table 1, split up the factors differently than in the DSM‐IV and King *et al*. (1998a) but found that the avoidance and numbing symptom clusters were not separate and actually included in a larger factor that they called dysphoria. Simms *et al*. (2002) used a clinician‐administered survey in a homogeneous sample of 3566 Gulf War veterans, and found that a four‐factor solution in a confirmatory factor analysis (CFA) fit better than a three‐ or two‐factor solution. Of note is the Simms *et al*. (2002) finding that the two avoidance items in the scale loaded on independent factors, but ultimately ended up including them as one in their reporting. Baschnagel *et al.* (2005) used a clinical sample of 528 undergraduate student 9/11 victims, Boelen *et al.* (2008) used a clinical sample of 347 bereaved mourners, Elklit and Shevlin (2007) used a clinical sample of 1116 people with a general history of PTSD, Krause *et al.* (2007) used a sample of 396 medical patients and 407 women seeking help for intimate partner violence, and Milanak and Berenbaum (2009) used a sample of 95 adults with trauma histories. Simms *et al*. (2002) identification of a dysphoria factor represented a departure from the DSM‐IV symptoms, as it spanned a category of items not previously associated with each other in the DSM‐IV or previous research.

Shevlin *et al.* (2009) compares King *et al.*'s (1998a) hyperarousal and Simms *et al.*'s (2002) dysphoria factors, as this discrepancy is the major difference between the two. They find evidence that Simms *et al.*'s (2002) dysphoria factor presents a better fitting model of PTSD. This is supported by Yufik and Simms' (2010) meta‐analysis, which found that both models are good candidates for modeling PTSD, but with a preference for the Simms *et al*. (2002) model. King *et al.* (2006) go so far as to say that there is little to no need for future exploratory factor analyses on the DSM‐IV, as these two four‐factor structures are so well established by the great wealth of research. They state that confirmatory analysis papers provide the best option for future researchers, specifically in the area of invariance and consistency of factor structures over gendered, racial, cultural subsets of trauma populations and over time utilizing longitudinal surveys. (Following up on this open thread in research, our work uses a unique dataset to investigate this invariability across respondents with heterogeneous levels of vulnerability in a large, diverse sample of disaster victims.)

Many studies using the self‐report DTS have confirmed the reliability of the scale outside of clinical settings. The DTS is typically administered when there is some reason that a clinician‐administered survey would be difficult to execute. All English‐language studies of the DTS include a reliability analysis, and have found that it is on the whole very reliable (Davidson *et al.*, 1997; McDonald *et al.*, 2009; Palmieri *et al.*, 2007). An additional established benefit of the DTS is how well its reliability holds up when administered in different languages. Translations of the DTS have, on the whole, held up remarkably well under reliability analysis. Chinese, Dutch, and Korean versions have each been independently assessed for reliability and found alpha values between 0.95 and 0.99 (Chen *et al.*, 2001; Declercq and Willemsen, 2006; Seo *et al.*, 2008).

Davidson's results also suggest that the composition of the sample can influence results of factor analyses of the scale. One area of trauma scale research in general and with respect to the DTS that remains under researched is how the factor structure is affected by using a large and heterogeneous sample. For example, heterogeneity in economic vulnerability or recovery environment can play a measurable role in the severity and persistence of psychological trauma (King *et al*., 1998b; Stallard *et al.*, 2001; Solomon and Mikulincer, 2006). The Chilean data contains a variety of post‐quake exposure and vulnerability measures for use in our concurrent validity analysis. A few studies have investigated the relationship of heterogeneous samples to the internal structure of trauma data, but few use self‐report trauma scales and most use small, homogenous samples. For instance, Naifeh *et al*. (2010) explores self‐report factors using the PTSD Checklist (PCL) scale in a clinically diagnosed sample of 407 Canadian Veterans and find, for example, different classes of symptom severity loaded heavily on Simms *et al*. (2002) factors of emotional numbing and dysphoria. Begić and Jokić‐Begić (2007), in a 151‐person sample of Croatian war victims using three combat‐specific self‐report PTSD scales (Mississippi Scale, Minnesota Multiphasic Personality Inventory, and Questionnaire on Traumatic Combat and War Experiences), finds that high‐intensity PTSD manifested itself in aggressive tendencies while low‐intensity PTSD manifested itself in depression and emotional symptoms. Switching back to applications specifically of the DTS, McDonald *et al.* (2008) assesses factorial invariance across three sets of veterans from three different wars. They note that while a four‐factor solution is commonly found in research, very little has been done to validate the invariance of factor solutions across samples that share a common traumatic event. They use three samples of 313 Operation Enduring Freedom/Operation Iraqi Freedom veterans, 814 Vietnam‐era veterans, and 320 Gulf War I veterans. They find a four‐factor solution in each one that aligns with King *et al*. (1998a) and demonstrate that these factors hold up across the three samples even though the wars were in different eras. Another example of studies assessing factorial invariance is Marshall (2004), who found that a four‐factor solution held up across English (299 person sample) – Spanish (120 person sample) language groups.

This paper directly addresses many of the open questions identified in this review of the literature. Because of the unique characteristics of the data, our research lent itself to satisfying some key research gaps in this area. Vulnerability measures facilitated a factor invariability analysis across different subsets of the data, and allowed for concurrent validity and alpha tests to be extended across those same subsets.

## Methods

### Participants and procedures

The application of the DTS used in this investigation comes from a panel design household survey gathered in 2009 and 2010 in the regions most affected by the 2010 Chilean earthquake. The survey was approved by the Observatorio Social de la Universidad Alberto Hurtado (OSUAH). The dataset, an extension of Chile's National Survey of Socio‐economic Characterization (CASEN) survey is available for free following the completion of an online form. Data is completely anonymous and informed consent was not deemed necessary for approval. The useable sample size, including respondents who were surveyed in both years and given all items used in the analysis, is 26,737. CASEN is a longitudinal questionnaire‐based survey distributed every two to three years across all of Chile's provinces, similar in structure to the United States American Community Survey (ACS). The last time the full survey was gathered was in 2009. In 2010, a supplemental survey was conducted a few months after the earthquake, contacting all households that had been included in 2009 and lived in the affected areas. These two surveys, with households and respondents mapped between the two periods, comprise the Post Earthquake Survey (EPT 2010). The final EPT 2010 questionnaire contains eight modules measuring a number of characteristics about a household. The EPT modules include control variables (disability, family status, age, etc.), education, work status, income and financial status, health, social capital, dwelling construction, and psychosocial impact. Extensive telephone, interview, and other procedures were undertaken to identify and distribute questionnaires to specific households and respondents who had moved between the two periods. This ensured that key data, like income loss, job loss, and whether or not someone had moved between the two periods, could be measured longitudinally.

The benefit of this data is that it fulfills all the characteristics of a dataset that could be used to fill the previously mentioned gaps in the use of survey instruments to measure the prevalence of psychological trauma. It is a large sample across a representative probabilistic cross‐section of Chilean society, it was gathered quickly, and it includes ways to distinguish between victims with different levels of exposure to the quake and varying physical and economic vulnerability.

### Measures

The dependent variable in this study is a respondent's score on the DTS. In this application, the administration of the scale was done in Spanish at an eighth grade reading level. The original Spanish‐language translation of the DTS was presented in Bobes *et al*. (2000). It was initially validated using convergent validity against four other Spanish‐ and English‐language scales and clinical data. Each of the 17 symptom questions from the DTS was asked twice, once for frequency and once for intensity of each symptom. Responses were given on a five‐point scale for frequency (0 = “not at all” to 4 = “every day”) and severity (0 = “not at all distressing” to 4 = “extremely distressing”), which were then added together for a maximum score of 8 for each symptom and an overall maximum score of 136. The frequency and intensity scores can also be interpreted independently giving respective subscales with a maximum value of 68, and the 17 symptoms can be further broken down into three symptom clusters that align with the clinical definition of psychological trauma symptom categories in the DSM‐IV. These three smaller scales can be interpreted independent of the overall and frequency/intensity scores to show if a person's traumatic condition is heavier on more specific trauma symptoms of hyperactivity, avoidance, etc.

The other variables measured in the EPT 2010 that are used in the analysis fit into three categories: economic volatility, poverty, and exposure. Two measures were used to assess economic volatility, both involving changes in economic status from before to after the quake. The first is a continuous measure of the difference in personal income between the periods in which the sample is divided into five quintiles drawn from the difference between pre‐ and post‐quake income. The second is an indicator of whether or not someone lost his or her job during the period.

Two discrete measures captured variations in respondents' personal poverty following the quake: crowded living quarters and income below the poverty line. The measure of crowded living quarters was based on the ratio of persons living in dwelling to beds with a ratio of less than 2.4 indicating no or low crowding, 2.5 to 4.0 a medium level, and greater than 4.0 critical. Whether or not a person was living below the poverty line in 2010 was determined by comparing reported income to the established national poverty line.

Two additional measures indicate exposure to the quake. Damage to a respondent's house was classified as low, medium, and high, based on illustrations in the survey manual. An independent measure of physical exposure to the traumatic event was obtained from seismologic data from the United States Geological Survey. We matched each respondent with intensity data (peak ground acceleration) at commune (third level administrative division) level. This was achieved by overlapping a vector map of the region with coordinates and intensity with a political map of Chile in ArcGIS. As with the income, this continuous measure was then broken into three terciles for subset analysis. Other spatial data like soil type or slope could have increased accuracy, but this commune measure was the most detailed exposure measure achievable with the data.

### Analysis procedures

To address the first research question, CFA using maximum likelihood estimation to analyze and compare the factor structure of the DTS in the Chile sample with clinical understandings of trauma and the structure of psychological trauma surveys as derived from the DSM‐IV. Data was complete within the 27,737 person sample and no sample weights were provided, outside of a restricted 16,086 person subsample of those with values for peak ground acceleration, so no steps were taken to account for missing data. We used a maximum likelihood (ML) estimation and examined the covariance matrix, based on their use in previous analyses of the DTS. We note that this is a methodological weakness due to a normality assumption in ML estimation (Mason *et al.*, 2013; Leiva‐Bianchi and Araneda, 2013). For more on this estimation method and matrix choice, see Brown (2006). No normality transformation was made on the data due to the necessary comparability and interpretability of the DTS scale scores. In preparation for the CFA, the 34 items in the DTS scale (symptom frequency and intensity) data were parceled into 17 overall measures, as is customary in other studies of this type.

King *et al*. (1998a) and Simms (2002) used exploratory factor analysis (EFA) to identify categories of scale items that are associated with each other. These particular four‐factor models have been confirmed multiple times in previous research, and the question of their validity has been largely answered. Davidson's two‐ and six‐factor models have largely been discredited due to small sample size, and were not included in the CFA. This investigation will start by repeating the analysis of previous studies in assessing the fit of the Chilean data's factor structure to these well‐established models. In order to assess the effect that a wide sample of Chilean earthquake victims has on factor structure, a CFA will also be conducted on a subset of the population who scored above a DTS threshold score of 40 (*n* = 3566), the group found in previous studies to most often have clinical diagnoses of PTSD. This is done to simulate the effect selection of a clinical trauma population in order to see if this characteristic offers any benefits to model fit. Reporting of CFA results, including choice of fit tests, comparative indices, and different methods of accounting for characteristics of a sample is a lengthy subject in itself. For more guidelines on reporting CFA results beyond what is discussed here, see Jackson *et al.* (2009). Our most important fit statistics are those that are able to account for different sample sizes and maximize the comparability of our results between subsets. Goodness‐of‐fit is measured by a number of statistics including a chi‐squared (*χ* ^2^) test, standard root‐mean‐squared residuals (SRMR) test, root‐mean‐squared error of approximation (RMSEA) test, Comparative Fit Index (CFI), and a relative non‐normed fit test, the Tucker–Lewis Index (TLI). A CFI test in particular is performed because it maximizes comparability by minimizing the effect of different sample sizes. It was chosen over *χ* ^2^ comparative tests like the Normative Fit Index (NFI) because NFI and CFI are indistinguishable in large samples and have a large negative bias in small samples (Bentler, 1990). We do not have any reason to assume statistical independence between the four factors, so we leave the covariance between our four factors unconstrained. The only constraint that we place on this CFA is the previously defined four‐factor categories. As such, the *χ* ^2^ tests for each model act as modification indices to determine which model presents the best fit overall when compared to the saturated single‐factor model. Standardized residuals will also be assessed to determine the extent to which model‐estimated item covariances differ from observed item covariances. A particular model and sample is chosen based on is overall and comparison best‐fit measures, and this model is used in further analysis.

CFA is further used to test the invariance of the factor structure across subgroups of the full sample differentiated by their levels of economic volatility, poverty, and exposure. Our invariance analysis assesses the fit of the best‐overall‐fit factor model across various volatility subsets of the population. By separating the data into subsets and running a grouped CFA, the investigation examines the extent to which the factor structure is invariant across samples with varying levels of economic volatility, poverty, and exposure. The fit of these models on the vulnerability subsets are assessed according to the same statistics as before. In this CFA invariance analysis, we assume that parameter means and factor loadings vary substantially across our subsets. By design, we chose subsets that displayed group‐specific response tendencies. For example, we can generally expect different mean levels of trauma across our vulnerability subsets. Thus, as in Gregorich (2006), constraining means would have resulted in differential additive response bias. As such, we did not see fit to impose any mean equality constraints. We should also note here that a result of this is that our subset invariance tests ended up being somewhat low on the hierarchy of invariance tests as detailed by Gregorich (2006). Ultimately, we made the choice to test invariance in the configuration of the factors (configural invariance) and invariance in the factor loads (metric invariance) across groups.

Cronbach's alpha was used to address the third research question regarding the reliability of the scale. Cronbach's alpha is a widely used measure of internal reliability, providing an estimate of the extent to which multiple items measure a single construct. Reliability is assessed for the total group and for the various subsets in the data.

To examine the fourth research question regarding the concurrent validity of the DTS, trauma scale scores are correlated with the collection of physical vulnerability, economic volatility, and exposure. Correlations between these variables and overall DTS scores are presented and discussed on the whole and among the previously defined subsets of the data. High concurrent validity would be indicated with strong correlations between the DTS and items directly related to the trauma of the quake and lower correlations with other measures.

## Results

Table 2 displays descriptive statistics, subsample breakdowns, and other socio‐economic breakdowns for all variables used in the analysis. Average DTS scores are shown for the total group of respondents and for each subsample. Results generally indicate that scores were higher for respondents suffering greater exposure and poverty (house damage, peak ground acceleration, house crowding, and poverty).

**Table 2:** Davidson Trauma Scale (DTS) descriptive statistics

	*n*	Score	Age	SD	Years of education	SD	Employed (%)
EPT 2010 sample	75,986	—	35.8	(22.4)	8.0	(4.8)	40.6
DTS sample	26,737	15.8	48.9	(17.6)	8.5	(7.9)	37.8
House damage	26,737	—	—	—	—	—	—
Low/none	23,641	14.0	51.0	(17.5)	8.6	(4.3)	38.7
Medium	2,200	27.2	50.3	(17.5)	7.7	(4.3)	33.0
High	896	34.4	48.7	(18.4)	7.1	(4.2)	28.3
House crowding	26,737	—	—	—	—	—	—
Low	23,465	15.4	50.1	(17.5)	8.5	(3.9)	38.1
Medium	2,986	17.7	40.5	(15.9)	8.3	(3.8)	33.0
Severe	286	24.7	43.9	(14.9)	7.8	(4.4)	28.3
Poverty (2010)	26,737	—	—	—	—	—	—
Yes	4,658	19.5	42.2	(15.2)	8.3	(3.8)	25.3
No	22,079	15.0	50.3	(17.7)	8.6	(4.3)	40.5
Lost job	25,844	—	—	—	—	—	—
No	23,247	16.4	49.9	(17.2)	8.3	(4.3)	42.2
Yes	2,597	15.9	46.1	(15.3)	8.6	(4.0)	0.0
Income loss quintile	26,737	—	—	—	—	—	—
Q1 (least loss)	5,407	14.3	49.6	(17.9)	8.9	(4.5)	50.0
Q2	5,372	16.6	48.9	(17.9)	8.0	(4.2)	48.3
Q3	5,269	17.3	48.0	(17.4)	8.1	(4.2)	46.6
Q4	5,297	16.0	48.0	(17.4)	8.2	(4.2)	47.2
Q5 (most loss)	5,392	14.8	50.1	(17.0)	9.1	(4.4)	48.6
PGA^a^	16,086	—	—	—	—	—	—
Tercile 1 (weak)	5,624	16.4	49.3	(17.8)	8.4	(4.2)	36.5
Tercile 2	5,205	25.6	49.7	(17.4)	7.9	(4.3)	34.6
Tercile 3 (strong)	5,257	29.6	50.2	(17.3)	8.2	(4.4)	34.5

a Smaller overall sample size on Peak Ground Acceleration (PGA) terciles.

Note: SD, standard deviation.

### Research question #1: confirmatory factor analysis (CFA)

**Figure 1:**
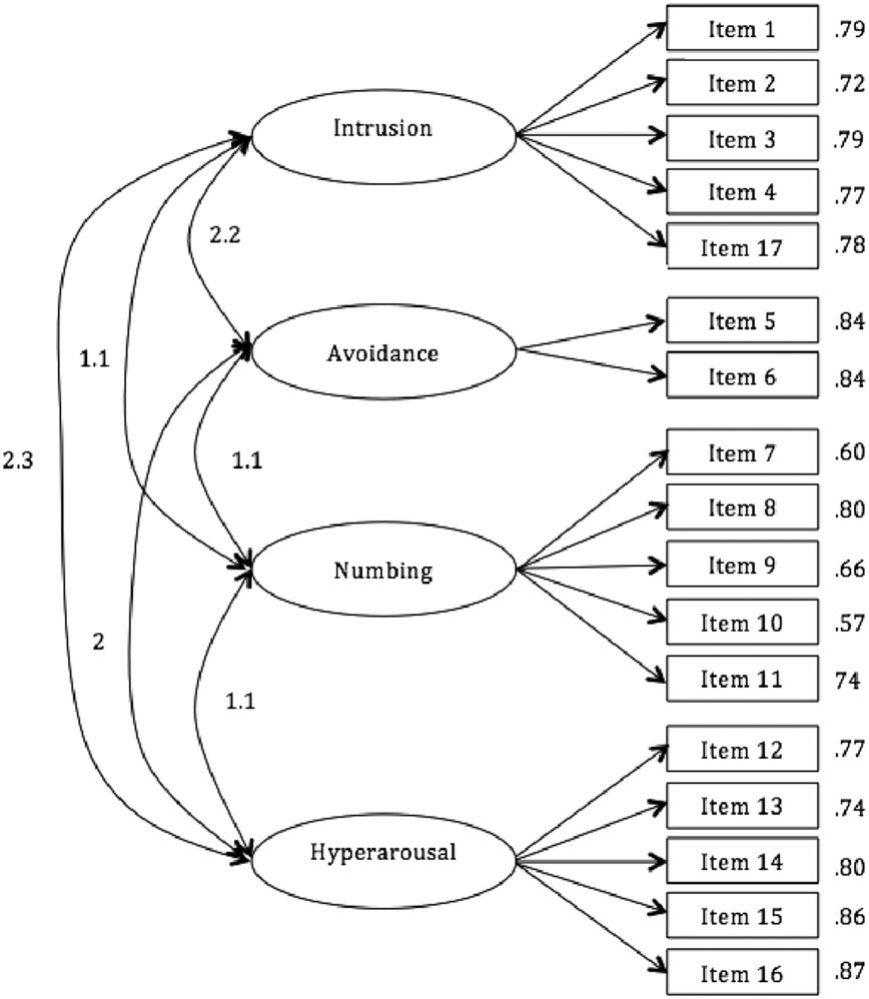
Full‐sample correlated four‐factor Davidson Trauma Scale (DTS) model from King *et al*. (1998a).

**Figure 2:**
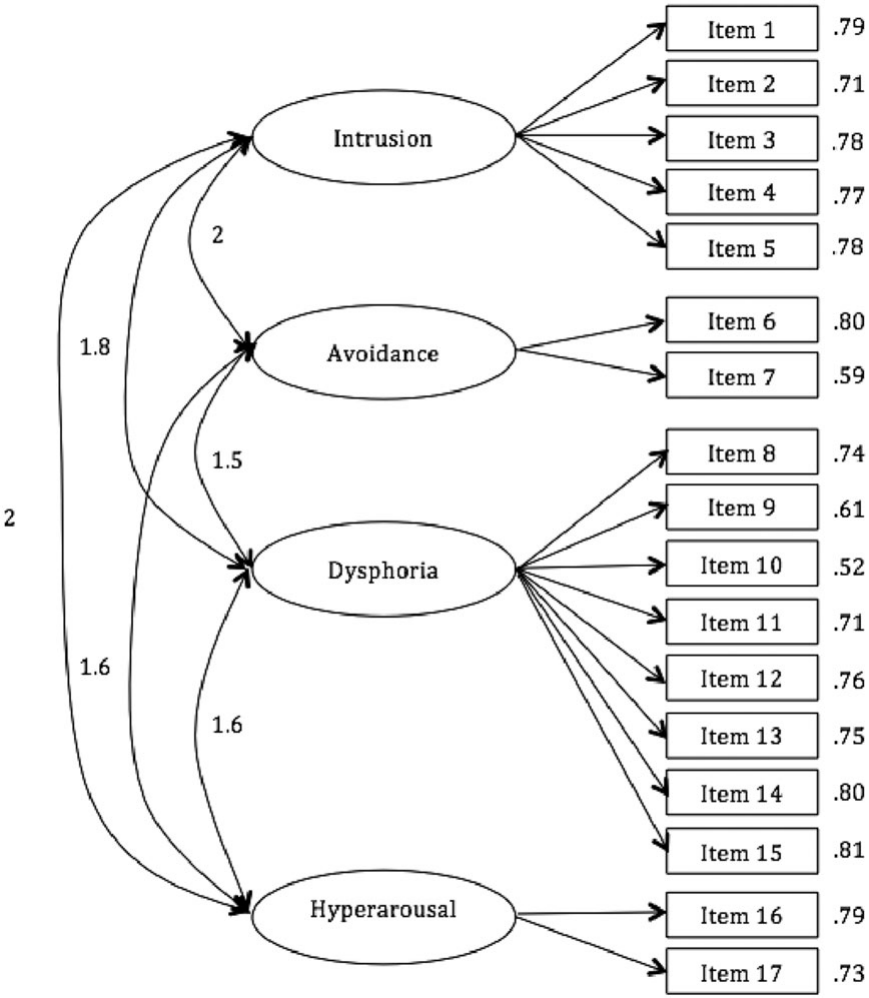
Full‐sample correlated four‐factor Davidson Trauma Scale (DTS) model from Simms *et al*. (2002).

Table 3 contains the statistical results of the initial CFA. Standardized factor loads are presented for each item in the groupings of the three potential models. The three potential models are the DSM‐IV groupings, King *et al*. (1998a) four‐factor solution, and Simms' (2002) four‐factor solution. Figures 1 and 2 show visual representations of the King *et al*. (1998a) and Simms (2002) models. For each model, the factor loads are presented for both the full sample and the 40‐threshold restricted diagnosis sample, both unconstrained by subset analysis. Below the factor loads are goodness‐of‐fit statistics. A good fit is one with high CFI and TLI (above 0.8), low RMSEA (ideally below 0.06), and low SRMR. Based on these measures, it is clear that a full sample fits these models much better than the restricted diagnosis sample, and that the King *et al*. (1998a) model fits slightly better than the DSM‐IV model and the Simms (2002) model. The RMSEA of this model which, in a large sample, is more reliable according to Rigdon (1996), is minimized and the CFI and TLI are maximized under the full sample King *et al*. (1998a) model. Examination of the standardized covariance residuals showed no evidence of problems stemming from incorrectly implied model item covariances at over a 99% confidence level in over 90% of total inter‐item covariances. Our modification index (*χ* ^2^) shows that while our reduced models understandably fit worse than the saturated model, the King *et al*. (1998a) model in general provides benefits to fit over the DSM‐IV and Simms *et al*. (2002) models. While both the Simms *et al*. (2002) provide acceptable fit statistics according to CFI and TLI, RMSEA is slightly high in both. Using the full sample instead of the high threshold sample also provided benefits to fit, although not among the modification index. For these reasons, we select the King *et al*. (1998a) model and a full sample as the best fit, and thus move on to further analysis using these selections.

**Table 3:** Initial confirmatory factor analysis (CFA) results

DSM‐IV theoretical				King *et al.* (1998a) model				Simms *et al.* (2002) model			
Category	Item #	Full sample	40‐Threshold	Category	Item #	Full sample	40‐Threshold	Category	Item #	Full sample	40‐Threshold
B	Item 1	0.79	0.56	Intrusive	Item 1	0.79	0.56	Intrusive	Item 1	0.79	0.56
	Item 2	0.71	0.55		Item 2	0.72	0.54		Item 2	0.71	0.52
	Item 3	0.79	0.63		Item 3	0.79	0.63		Item 3	0.78	0.61
	Item 4	0.77	0.6		Item 4	0.77	0.61		Item 4	0.77	0.61
	Item 17	0.78	0.57		Item 17	0.78	0.57		Item 5	0.78	0.53
C	Item 5	0.78	0.55	Avoidance	Item 5	0.84	0.68	Avoidance	Item 6	0.8	0.67
	Item 6	0.79	0.6		Item 6	0.84	0.73		Item 7	0.59	0.35
	Item 7	0.6	0.39	Numbing	Item 7	0.6	0.41	Dysphoria	Item 8	0.75	0.47
	Item 8	0.78	0.56		Item 8	0.8	0.59		Item 9	0.61	0.42
	Item 9	0.62	0.41		Item 9	0.66	0.53		Item 10	0.52	0.33
	Item 10	0.54	0.35		Item 10	0.57	0.47		Item 11	0.7	0.43
	Item 11	0.72	0.43		Item 11	0.74	0.5		Item 12	0.76	0.47
D	Item 12	0.77	0.43	Hyper‐arousal	Item 12	0.77	0.44		Item 13	0.75	0.53
	Item 13	0.73	0.41		Item 13	0.74	0.42		Item 14	0.8	0.63
	Item 14	0.8	0.52		Item 14	0.8	0.53		Item 15	0.82	0.57
	Item 15	0.87	0.8		Item 15	0.86	0.79	Hyper‐arousal	Item 16	0.73	0.47
	Item 16	0.87	0.77		Item 16	0.86	0.76		Item 17	0.79	0.47
Fit	*χ* ^2^	16,275	2,742	Fit	*χ* ^2^	12,186	2,054	Fit	*χ* ^2^	23,093	3,851
	SRMR	0.034	0.064		SRMR	0.029	0.055		SRMR	0.038	0.070
	RMSEA	0.072	0.080		RMSEA	0.063	0.069		RMSEA	0.087	0.096
	CFI	0.94	0.80		CFI	0.96	0.85		CFI	0.92	0.71
	TLI	0.94	0.76		TLI	0.95	0.82		TLI	0.90	0.65

### Research question #2: structural invariance across subsets

We proceed to test configural invariance first by dividing the full sample into subsets indicating economic volatility, personal poverty outcomes, and exposure and running single‐group confirmatory factor analyses using the selected King *et al*. (1998a) factor model. The results are presented in Table 4. The standardized factor loads are excluded from these CFA results for simplicity in favor of the goodness‐of‐fit statistics. Single‐group fit statistics are somewhat invariant across the economic volatility indicators of income loss and job loss, with a minimum of 0.95 CFI and maximum RMSEA of 0.068. These results are similar across the personal poverty outcome variable indicating poverty in 2010, and the exposure variable for peak ground acceleration. However, note the interesting fit results on house crowding and damage. That is, the RMSEA goes from 0.062 to a worse 0.083 from low to high crowding, and 0.062 to 0.077 from low to high damage. SRMR peaks at 0.49 in the high crowding subset, and 0.038 in the high damage subset. CFI and TLI mirror these changes, with CFI and TLI at a sample‐low 0.92 and 0.90 in high crowding. When compared to how much the fit statistics changed in the rest of the subsets, we view these differences in CFI, TLI, SRMR, and RMSEA as significant. Where economic volatility seems to have little effect on the fit of these models, the variables for physical vulnerability seem to have a measurable negative effect on the goodness‐of‐fit in these subsets.

**Table 4:** Subset breakdown CFA results

*Ecomomic volatility variables*							
Goodness‐of‐fit statistics	Income loss quintiles					Lost job	
	1	2	3	4	5	Yes	No
Obs	5,407	5,372	5,269	5,297	5,392	2,597	23,247
χ2	2,665	2,618	2,854	2,444	2,926	8,109	4,284
SRMR	0.030	0.029	0.031	0.029	0.030	0.030	0.027
RMSEA	0.065	0.064	0.068	0.062	0.068	0.066	0.061
CFI	0.95	0.96	0.95	0.96	0.95	0.96	0.96
TLI	0.94	0.95	0.94	0.95	0.95	0.95	0.95

*Personal poverty outcome variables*						
Goodness‐of‐fit statistics	Crowding			Poverty (2010)			
	Low	Medium	High	Yes	No		
Obs	23,458	2,981	286	4,658	22,079		
χ2	10,440	1,888	391	2,334	10,089		
SRMR	0.028	0.033	0.049	0.030	0.029		
RMSEA	0.062	0.073	0.083	0.065	0.063		
CFI	0.96	0.95	0.92	0.96	0.96		
TLI	0.95	0.94	0.90	0.95	0.95		

*Exposure variables*						
Goodness‐of‐fit statistics	Peak ground acceleration tercile^a^			Damage			
	Low	Medium	High	Low	Medium	High	
Obs	5,624	5,205	5,257	23,641	2,200	896	
χ2	2,509	2,758	2,639	10,303	1,329	792	
SRMR	0.028	0.033	0.033	0.029	0.032	0.038	
RMSEA	0.061	0.067	0.065	0.062	0.070	0.077	
CFI	0.96	0.95	0.96	0.96	0.95	0.94	
TLI	0.95	0.94	0.95	0.95	0.94	0.93	

a Only 16,086 of overall 27,737‐person sample had entries for peak ground acceleration.

A summary of the configural and metric invariance tests are presented in Table 5. Metric invariance was not confirmed across any of the subsets using a *χ* ^2^ difference test. This *χ* ^2^ difference test was performed between two candidate models for each grouping. In the first model, factor loads were allowed to float freely across the groups. In the second, factor loads were constrained to be equal. We found evidence that the model with factor constraints provided significantly worse fit across all groups, and thus reject the hypothesis of metric invariance.

**Table 5:** Summary of configural and metric invariance tests

Subset/model	*χ* ^2^Δ	Δdf	*p*	Invariant
Income loss				
Configural invariance	—	—	—	Yes
Metric invariance	186.46	52	<0.001	No
Lost job				
Configural invariance	—	—	—	Yes
Metric invariance	46.73	13	<0.001	No
Crowding				
Configural invariance	—	—	—	Yes
Metric invariance	98.74	26	<0.001	No
Poverty				
Configural invariance	—	—	—	Yes
Metric invariance	98.87	13	<0.001	No
Peak ground acceleration (restricted sample)				
Configural invariance	—	—	—	Yes
Metric invariance	175.9	26	<0.001	No
Damage				
Configural invariance	—	—	—	Yes
Metric invariance	436.97	39	<0.001	No

### Research question #3: Cronbach's alpha reliability across subsets

Cronbach's alpha reliability measurements are shown in Table 6. The value of 0.95 for the total scale indicates a very high level of internal consistency and is very similar to other non‐English studies that included the DTS (Chen *et al.*, 2001; Declercq and Willemsen, 2006; Seo *et al.*, 2008). Alpha estimates for subsets of the population are also shown in Table 6. These results show that the scale is not only reliable as a whole, but that we can find high internal consistency across subsets of the population, with all values falling between 0.94 and 0.95.

**Table 6:** Cronbach's alpha comparisons/data subsets

Study		Sample		*n*	Cronbach's alpha
This study		Earthquake victims (Spanish)		26,737	0.95
Davidson *et al*. (1997)		Rape, war, hurricane victims (USA)		241	0.99
Chen *et al.* (2001)		Earthquake victims (Chinese)		210	0.97
Declercq and Willemsen (2006)		Security company and Red Cross (Belgium)		544	0.97
Ford‐Gilboe *et al.* (2009)		Domestic abuse victims (Canada)		309	0.95
Seo *et al.* (2008)		PTSD patients (Korea)		254	0.97

Data subset	*n*	Cronbach's alpha	Data subset	*n*	Cronbach's alpha
Income loss			House crowding		
Q1	5,407	0.943	None	23,458	0.946
Q2	5,372	0.946	Medium	2,981	0.950
Q3	5,269	0.947	Severe	286	0.947
Q4	5,297	0.947			
Q5	5,392	0.949	House damage		
			Low/none	23,641	0.944
Below poverty line (2010)			Moderate	2,200	0.944
No	22,079	0.946	Heavy	896	0.954
Yes	4,658	0.949			
			Peak ground acceleration tercile^a^		
Lost job			T1	5,624	0.941
No	23,247	0.946	T2	5,205	0.942
Yes	2,597	0.950	T3	5,257	0.945

a Only 16,086 of overall 27,737‐person sample had entries for peak ground acceleration.

### Research question #4: concurrent validity of the DTS across subsets

Table 7 gives the results of our concurrent validity analysis, displaying pairwise correlations between overall DTS scores and the collection of economic volatility, personal poverty outcome, and exposure variables. The results were calculated using the full range of DTS scores to provide maximum variability. The first line gives the results for the total group; and the following lines give results for each of the subgroups. There was a very clear ordering in the effect that each of these variables had on trauma scores. Housing damage on the whole had the largest correlation with scores, at 0.29. Peak ground acceleration, another physical vulnerability measure, was second at 0.19. The rest of the correlations were low to none, with poverty in 2010 being the highest of the remaining variables at 0.08 and income loss quintile overall uncorrelated.

**Table 7:** Pairwise concurrent validity correlations

DTS correlation and subset	Peak ground acceleration	House damage	House crowding	Poverty (2010)	Lost job	Income loss quintile
Overall sample	0.19	0.29	0.05	0.08	0.01	0.00
House damage						
Low/none	0.18	—	0.01	0.06	0.01	0.00
Medium	0.10	—	0.01	0.02	−0.01	−0.02
High	−0.06	—	−0.02	0.04	0.00	0.04
House crowding						
Low	0.19	0.28	—	0.07	0.01	0.00
Medium	0.19	0.29	—	0.07	0.01	0.00
Severe	0.19	0.28	—	0.07	0.01	0.00
Poverty (2010)						
Yes	0.18	0.27	0.02	—	−0.02	−0.03
No	0.20	0.29	0.04	—	0.01	0.01
Lost job						
Yes	0.20	0.29	0.06	0.06	—	−0.03
No	0.19	0.29	0.05	0.08	—	0.00
Income loss quintile						
Q1	0.20	0.28	0.03	0.06	0.02	—
Q2	0.17	0.29	0.05	0.05	0.00	—
Q3	0.22	0.30	0.04	0.06	0.04	—
Q4	0.19	0.29	0.04	0.09	−0.01	—
Q5	0.19	0.29	0.05	0.10	0.01	—

## Discussion

The value of a relatively easily administered survey instrument to measure trauma incidence and severity is clear. Such a survey could be given to a large random sample of an affected population, with data gathered cheaply through self‐reporting or interviews with non‐specialists. Next, in this particular survey, we were mainly limited by availability of data and changing understanding of and research into PTSD and its symptoms.

It is not surprising that metric invariance was not supported by this analysis using this data. Previous studies that have tested metric invariance typically do so with the initial hypothesis that factor loads *are* equal across subsets of very similar trauma patients. McDonald *et al.* (2008) for example, tested and confirmed metric invariance across subsets of veterans in different wars. These types of studies search for similarities in factor structure between similar groups of trauma victims. However, our results spanned a large section of Chilean society, and our subsets were not chosen with the assumption of comparability. In fact, the rejection of metric invariance at a surface level opens further questions about how factor load patterns of trauma *are* more specifically affected by these economic and social vulnerabilities.

The strength of this analysis could presumably have been augmented by the extension of the concurrent validity analysis to other trauma symptom scales measured in parallel to the DTS (convergent validity). These scales could yield higher correlations with DTS scores than the measures included and would strengthen this particular argument. But these scales were not included in the survey, and this sort of analysis could not be performed. More data on the extended recovery environment of respondents would have also helped this analysis. For example, the data spanned two time periods, which lent itself well to measuring economic *volatility* through the disaster. But without more time periods, there were limited ways to test how economically *resilient* respondents were over time. Other data, like participation in social services and other quality of life variables were frustratingly incomplete and limited our sample too much to justify their inclusion.

Next, the understating of PTSD is a constantly‐changing field. The qualitative description of psychological disorders used in this paper is at this point somewhat outdated. The recently published fifth version of the DSM updates the definitions of PTSD to include new causes and symptoms in accordance with current research on traumatic disorders. For example, research showed that the anxiety symptom criteria from the DSM‐IV have very little predictive power in delivering a clinical PTSD diagnosis. The DSM‐V reflects this research by reclassifying PTSD from an anxiety disorder to a stress‐ or trauma‐related disorder, removing the anxiety symptom criteria, and adding new behavioral symptom criteria. New surveys will have to eventually be published and validated to account for these changes, with new factor structures potentially identified. However, the latest structure is still very new, and there is a much greater availability of survey structures, research and data derived from the DSM‐IV, which was used from 1994 to 2013. This study is still important, however because it satisfies a key research question that went largely unfulfilled in the DSM‐IV research, and will not be able to be explored again until a similar survey, with these unique characteristics, is produced using the DSM‐V.

## Conclusion

We set out in this investigation to confirm the validity of this application of the DTS to a large, heterogeneous population of earthquake victims in Chile. Through various techniques, we found that the data from the EPT 2010 in Chile is, on the whole, remarkably reliable and with an underlying factor structure that strongly confirms a previously established factor structure.

Concurrent validity was confirmed by moderate correlations between overall DTS scores and variables usually associated with higher trauma, such as house damage and strength of the trauma, and low or zero correlations with other variables. In addition, Cronbach's alpha values confirmed the scale's consistency. All of these results held up across different subsets of the data.

CFA allowed us to select a best‐fitting model, one from King *et al*. (1998a), to use in our invariability analysis, and this led to interesting results. Factor structure was invariant across our economic volatility subsets, but evidence of a breakdown in configural invariance was found when we tried to confirm the scale among mutually exclusive crowding and house damage subsets. Metric invariance was not supported among any subset. The full scale was found to be highly reliable, with Cronbach's alpha values of 0.94 or higher for the total sample and the previously mentioned subsets of the data. This CFA provided an interesting result, because it somewhat answers a question that has been raised in previous research while leaving some crucial questions about trauma scale variability open to future researchers. The evidence of the variability found here is weak, but hard to ignore. Because of the rather limited nature of this data, we leave this thread open to future researchers. Data targeted to a specific measurement, like long‐term recovery environment, social support system, or other characteristics certainly would facilitate this more extensive analysis.

## Declaration of interest statement

The author has no competing interests.
